# Clinical and Laboratory Features of PCR-Confirmed and Clinically Suspected COVID-19 Pediatric Patients: A Single Hospital-Based Experience During the First COVID-19 Wave in the United Arab Emirates

**DOI:** 10.3389/fped.2022.830587

**Published:** 2022-03-15

**Authors:** Nashwa M. B. Eldin, Maysa Saleh, Bahaaeldin Labib, Marwa Othman, Lalu Chacko, Daphne Mae, Lamiaa Elnour, Rami H. Al-Rifai

**Affiliations:** ^1^Department of Pediatric, Burjeel Hospital, Abu Dhabi, United Arab Emirates; ^2^Pediatric Department, Al Jalila Children's Specialty Hospital, Dubai, United Arab Emirates; ^3^Zayed Military Hospital, Abu Dhabi, United Arab Emirates; ^4^Burjeel Hospital, Abu Dhabi, United Arab Emirates; ^5^Institute of Public Health, College of Medicine and Health Sciences, Al Ain, United Arab Emirates

**Keywords:** pediatrics, children, COVID-19, SARS-CoV-2, United Arab Emirates

## Abstract

**Objective:**

This study investigated clinical and laboratory differences between confirmed (RT-PCR-positive) and clinically suspected (RT-PCR-negative) COVID-19 pediatric patients, and explored factors associated with disease severity at presentation and duration of hospitalization.

**Methods:**

Medical charts of COVID-19-confirmed and clinically suspected pediatric patients admitted to a tertiary hospital in Abu Dhabi were reviewed. Sociodemographic information and clinical and laboratory outcomes were retrieved and analyzed.

**Results:**

Between 1 April to 30 June, 2020, 173 patients (mean age: 3.6 ± SD 3.2 years) presented with respiratory symptoms. Of them, 18.0% had confirmed contact with COVID-19 cases, 66.5% had symptoms for ≤3 days, and 86.7% were with moderate to severe disease. Twenty-eight (16.1%) patients tested positive while the rest (83.8%) tested negative in RT-PCR. COVID-19-confirmed and clinically suspected patients were statistically similar (*p* > 0.05) in all sociodemographic data, disease severity, and vital signs except residence status (89.3% vs. 58.6% were residents, respectively, *p* = 0.002) and contact with confirmed COVID-19 cases (82.1% vs. 5.5%, respectively, *p* < 0.001). Fever (100 and 91.0%) and cough (100 and 95.9%) were the most common symptoms in both confirmed and clinically suspected COVID-19 patients. All patients were statistically comparable in mean white blood cell and platelet counts and hemoglobin concentration, except in mean concentration of neutrophils (higher in clinically suspected, *p* = 0.019). C-reactive protein was two times higher in clinically suspected compared to confirmed patients (*p* = 0.043). Lymphocyte (OR: 1.31, *p* < 0.001), LDH (OR: 1.01, *p* = 0.001), D-dimer (OR: 1.92, *p* < 0.001), and ferritin levels after 24–36 h (OR: 9.25, *p* < 0.05), and SGPT (OR: 1.04, *p* < 0.05) were all associated with disease severity. Elevated ferritin (>300 μg/L) after 24–36 h was the only correlated factor with disease severity (aOR: 17.38, *p* < 0.05). Confirmed compared with clinically suspected patients (aOR: 4.00, 95% CI: 2.92–5.10) and children with moderate compared with mild disease (aOR: 5.87, 95% CI: 1.08–32.06) had longer hospitalization.

**Conclusion:**

In pediatric patients with negative RT-PCR, COVID-19 is still suspected based on clinical symptoms and epidemiological data. A tentative diagnosis can be made based on a thorough examination, and proper medical management can be initiated promptly.

## Introduction

In December 2019, the first case of a novel coronavirus strain (2019-nCoV) was reported in China (1). One month later, the World Health Organization (WHO) announced 2019-nCoV, later known as SARS-CoV-2, as a global pandemic and it was termed coronavirus disease 2019 (COVID-19). The pandemic created a health and economic crisis that prompted the medical and scientific communities worldwide to solve the puzzle of this virus and its consequences. Since the early phase of the pandemic, the prevalence and clinical manifestations of COVID-19 were found more in adults and elderly populations compared to children (2–4). Recent reports documented that compared with adults, COVID-19 infection in children is less severe (5, 6).

In the United Arab Emirates (UAE), the first PCR-confirmed case of COVID-19 was reported on 29 January, 2020 (7). Since then, all efforts were put into understanding the disease characteristics, with massive testing, contact tracing, non-pharmaceutical interventions, new therapeutic modalities, and vaccination; all playing a crucial role in containing the spread of COVID-19 infection in the UAE (7). In Abu Dhabi, the capital and the largest city in the UAE, 43% of the first diagnosed cohort of the COVID-19-confirmed cases were asymptomatic (7). Although reports demonstrated that COVID-19 infection is less severe in children compared to adults—it includes mainly mild or moderate infections (2, 4–6)—yet, severe illness due to COVID-19 can occur in children, requiring hospitalization and intensive care (8). Originally, the incidence of COVID-19 in children younger than 18 years was 2.1% with a mortality rate of 0.2% (9). With the progress of the disease, evolving of different strains, and subsequent waves of COVID-19 infection, indicators of severe disease among hospitalized children with the Delta variant were generally similar to those observed earlier in the pandemic (10).

The diagnosis of COVID-19 is initially made on the basis of the patient's history and clinical features that are also supported by the real-time reverse transcriptase polymerase chain reaction (RT-PCR) diagnostic panel, which remains the standard for diagnosing COVID-19. However, an initial RT-PCR may still display a false-negative result (11), which increases the risk of community transmission and delay in treatment, particularly among asymptomatic individuals. Given that SARS-CoV-2 manifestations in children often resemble other high-prevalence viral infections in childhood, it is worth evaluating differences in clinical characteristics of confirmed (RT-PCR-positive) and clinically suspected (RT-PCR-negative) COVID-19 pediatric patients.

International reports of COVID-19 infections in children have increased steadily, but studies on the disease characteristics in the Middle East and UAE pediatric populations are noticeably limited. To the best of our knowledge, this study will be the first in the UAE and the Middle Eastern region to conduct a comparison between confirmed and clinically suspected COVID-19 pediatric patients admitted at one of the tertiary hospitals, the Burjeel Hospital, which became a designated facility to manage COVID-19 patients in Abu Dhabi, UAE.

This study aimed at understanding the clinical and laboratory differences between RT-PCR-confirmed and clinically suspected COVID-19 pediatric patients. Also, the study explored factors associated with disease severity at presentation and over the duration of hospitalization in pediatric patients.

## Materials and Methods

### Data Source and Extraction

Medical charts of all COVID-19-treated pediatric patients who were admitted to the Burjeel Hospital in Abu Dhabi, UAE, during the period between March and June 2020 were reviewed. At the time of presentation, information on patient's age, gender, residence status, history of contact with confirmed COVID-19 cases, history of travel in the past month, duration of symptoms, vital signs [body temperature, heart rate, respiratory rate, oxygen saturation (SpO2)], and physical examination was documented. Information on imaging (chest X-ray and or CT scan) and laboratory tests (full blood count, inflammatory and liver function biomarkers), medications (oxygen requirement, antibiotic use, antiviral use, bronchodilators use), and clinical outcomes was also extracted from medical charts. Information on the RT-PCR testing results was also extracted and documented as positive or negative. In this study, pediatric patients who tested positive to COVID-19 by RT-PCR were labeled as confirmed while pediatric patients who tested negative were labeled as clinically suspected COVID-19 patients.

### Data Management and Analysis

Data were extracted by an expert nurse to a pre-defined Excel sheet. Age in months and years was extracted then categorized into two groups (≤3 years or >3 years). Residence status was categorized as citizen or resident. Contact with confirmed COVID-19 cases (yes or no), travel history (yes or no), and duration of symptoms (≤3 days or >3 days) were all reported as dichotomous variables. Following the National Institute of Health guidelines (8) as well as internationally agreed disease severity at presentation was categorized into mild, moderate, severe, or critical. Mild illness was considered in cases that presented any apparent COVID-19-related symptoms (e.g., fever, cough, sore throat, malaise, headache, muscle pain) without shortness of breath, dyspnea, or abnormal imaging. Moderate illness was considered in cases presenting with lower respiratory diseases by clinical assessment or imaging and an oxygen saturation (SaO2) over 93% on room air. Severe illness was considered in cases presenting with a respiratory rate of over 30 breaths per minute and an SpO2 up to 93% at room air. Critical illness was considered in cases with a rapid disease progression in particular those who developed respiratory failure with the need for mechanical ventilation (i.e., acute respiratory distress syndrome, persistent hypoxia), septic shock, or multiple organ failure.

Blood profiles comprised the count of leukocytes, neutrophils, lymphocytes, and platelets, and hemoglobin concentration. Measured inflammatory biomarkers were the C-reactive protein (CRP) (normal ≤ 4 mg/L or elevated > 4 mg/L), procalcitonin (PCT) (normal: <0.1 ng/dl), LDH (normal (≤ 250 U/L or elevated > 250 U/L), ferritin level at presentation and after 24-36 h (normal ≤ 300 μg/L or elevated > 300 μg/L), and D-dimer (normal ≤ 1 mg/L or elevated > 1 mg/L), in addition to their continuous values. Based on these measured inflammatory biomarkers, pediatric patients were categorized into six groups (none elevated, only one elevated, only two elevated, only three elevated, and all elevated, or at least one elevated vs. none elevated). This categorization took into consideration the measured ferritin level after 24–36 h. The change in the ferritin level after 24–36 h compared to the ferritin level at presentation was quantified, then the ferritin level was considered either doubled or not. Moreover, to quantify the magnitude of change in the ferritin level, the ratio between the ferritin level at 24–36 h and at presentation was quantified. Findings from the chest X-ray and/or CT scan were categorized into three groups (normal, mild abnormalities, major abnormalities). Oxygen requirement (yes or no), antibiotic use (single combination), bronchodilators use (yes or no), and duration of hospitalization (≥3 days or >3 days) each were categorized into two groups. The exact antiviral used was also documented. Clinical outcome was documented as either needing regular care, high-dependency care, admitted to pediatric ICU, or death.

Continuous variables were reported as mean and standard deviation (SD). Between the RT-PCR-positive and RT-PCR-negative pediatric patients, means of the continuous variables were compared using the independent samples *t*-test. The assumption of equal variance was considered when the *p*-value to assess the mean difference between the two groups was recorded. Frequency and proportion of the categorical variables were reported. The difference in the proportion of patients in the measured categorical variables was examined using the Chi-square test. The *p*-value examining the difference in the proportion was extracted from the Pearson Chi-square test or from the Fisher's exact test, whenever applicable.

To explore associated factors, including positivity status in the RT-PCR test, with severity of the disease (severe vs. mild-moderate) at presentation and with duration of hospitalization as a continuous and as a dichotomous variable (≥3 vs. <3 days), bivariable and multivariable logistic regression analyses were performed. In the multivariable model, all factors showing a significant association at *p*-value <0.05 in addition to the positivity status in the RT-PCR test were included. Crude odds ratio (OR) and adjusted OR (aOR) with their 95% confidence intervals (95% CIs) were reported.

Data analysis was performed using IBM SPSS version 26. *P*-value <0.05 was considered indicative to significant difference. This study followed and was reported according to the Strengthening the Reporting of Observational Studies in Epidemiology (STROBE) reporting guidelines.

## Results

### Sociodemographic Information, Vital Signs, and Symptoms at Admission

During the study period, 173 children presented to the pediatric department with clinical presentation of COVID-19. The age of patients ranged from 3 months to 14 years (mean age: 3.6 ± SD 3.2 years). Nearly two-thirds (63.6%) of patients were residents, 18.0% reported a history of contact with a confirmed COVID-19 case, two-thirds (66.5%) reported symptoms ≤3 days, and 86.7% were in a moderate to a severe disease stage (Table 1).

**Table 1:** Distribution of confirmed (RT-PCR-positive) and clinically suspected (RT-PCR-negative) COVID-19 pediatric patients according to their sociodemographic and medical characteristics factors associated with RT-PCR-positive patients.

**Characteristics**	**All** ***n* = 173 (valid %)**	**Confirmed COVID-19** ***n* = 28 (valid %)**	**Clinically suspected** ***n* = 145 (valid %)**	***P*-value**	**OR (95% CI)**
**Age** (range: 3.0 months−14 years)	43.5 ± 38.2	47.3 ± 46.9	42.8 ± 36.4	0.564	1.04 (0.92–1.17)
Months ± SD (year ± SD)	(3.6 ± 3.2)	(3.9 ± 3.9)	(3.6 ± 3.0)	0.629	1.00 (0.99–1.01)
≤ 3 years	107 (61.8)	17 (60.7)	90 (62.1)	0.526	1.00
>3 years	66 (38.2)	11 (39.3)	55 (37.9)		1.06 (0.46–2.43)
**Gender**				0.069	
Male	89 (51.4)	10 (35.7)	79 (54.5)		1.00
Female	84 (48.6)	18 (64.3)	66 (45.5)		2.16 (0.93–4.99)
**Residence status**				0.002	
Citizen	63 (36.4)	3 (10.7)	60 (41.4)		1.00
Resident	110 (63.6)	25 (89.3)	85 (58.6)		5.88 (1.70–20.4)
**Contact to COVID-19 case**				<0.001	
No	112 (82.1)	5 (17.9)	137 (94.5)		1.00
Yes	31 (17.9)	23 (82.1)	8 (5.5)		78.8 (23.7–261.9)
**Travel**				NA	
Yes	0 (0.0)	0 (0.0)	0 (0.0)		
No	173 (100)	28 (100)	145 (100)		
**Duration of symptoms**				0.481	
≤ 3 days	115 (66.5)	17 (60.7)	98 (67.6)		1.00
>3 days	58 (33.5)	11 (39.3)	47 (32.4)		1.35 (0.58–3.11)
**Disease severity**				0.553	
Mild	18 (10.4)	2 (7.1)	16 (11.1)		
Moderate	117 (67.6)	22 (78.6)	95 (66.0)		
Severe	33 (19.1)	4 (14.3)	29 (20.1)		
Undefined mild-moderate	4 (2.3)	0 (0.0)	4 (2.8)		
*Missing*	1 (0.6)		1		
**Vitals on admission**
Mean body temperature ± SD	37.99 ± 1.1	37.7 ± 0.98	38.1 ± 1.01	0.085	
Mean heart rate ± SD	129.5 ± 21.3	122.9 ± 23.0	130.8 ± 20.8	0.072	
Mean respiratory rate ± SD	27.5 ± 6.4	25.8 ± 4.8	27.8 ± 6.7	0.142	
Mean SpO2 ± SD	98.6 ± 1.33	98.3 ± 1.46	98.7 ± 1.30	0.220	

*SD, standard deviation*.

Of the 173 pediatric patients, 28 (16.1%) were confirmed while the rest (83.8%) were clinically suspected COVID-19 patients (Table 1). Of the 28 confirmed COVID-19 patients, 60.7% were ≤3 years old, 64.3% were females, the majority (89.3%) were residents, 82.1% reported a history of contact with a confirmed COVID-19 case, 60.7% reported symptoms within the past ≤3 days, and 78.6% had a moderate disease status. Confirmed and clinically suspected COVID-19 pediatric patients were statistically similar in all of the measured sociodemographic characteristics, severity of the disease, and vital signs except in the status of residence (*p* = 0.002) and history of contact to a confirmed COVID-19 case (82.1% vs. 5.5%, respectively, *p* < 0.001) (Table 1).

**Figure 1:**
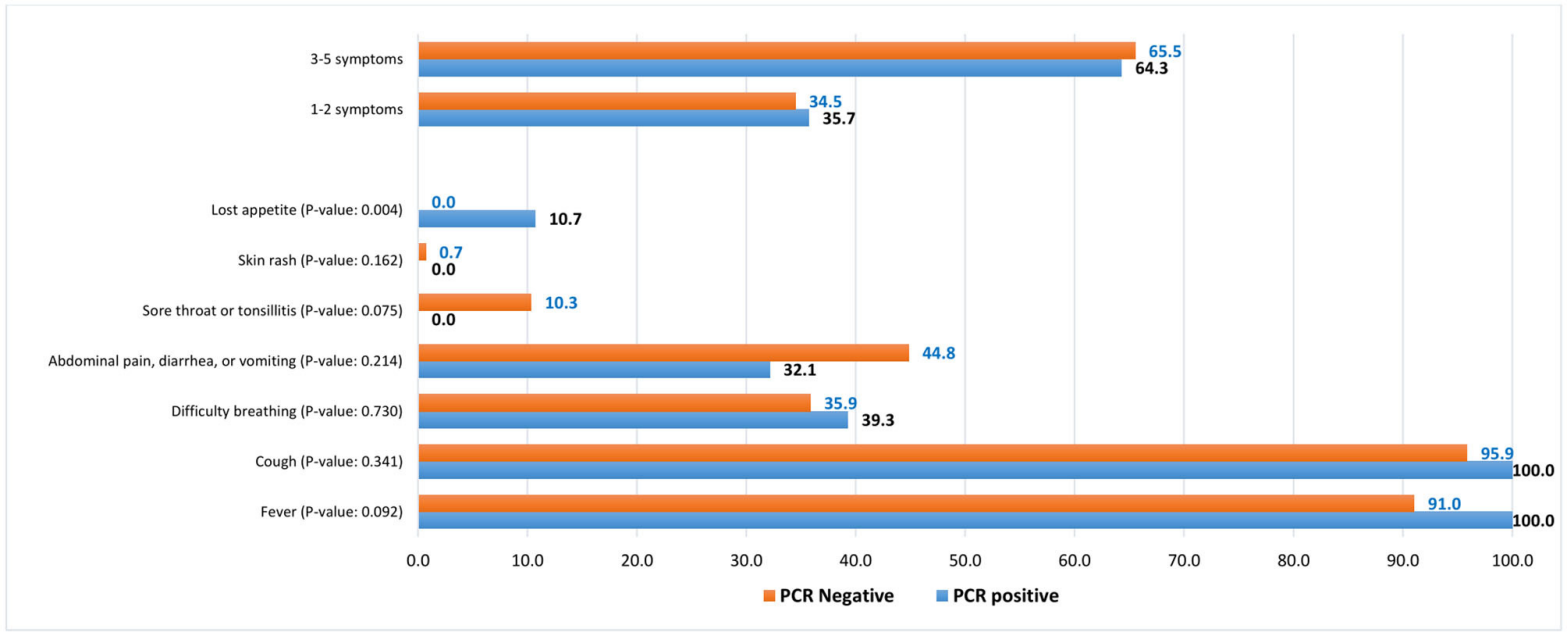
Symptoms distribution among COVID-19 RT-PCR-positive and clinically suspected COVID-19 (RT-PCR-negative) pediatric patients, %.

There was also an insignificant difference in the reported symptoms by COVID-19 status. The most common symptoms were fever (100% vs. 91.0%) with a comparable mean body temperature (37.7°C vs. 38.1^o^C in suspected, *p* = 0.085), cough (100% vs. 95.9%), difficulty in breathing (39.3% vs. 35.9%), and abdominal pain, diarrhea, or vomiting (32.1% vs. 44.8%), in confirmed vs. suspected COVID-19 patients, respectively (Figure 1).

### Blood Work and Chest X-Ray Examination

In all patients, the mean level of white blood cells (leukocytes and lymphocytes), platelets count, and hemoglobin concentration was within the normal range, with a statistically insignificant difference between COVID-19 confirmed and clinically suspected pediatric patients. The mean concentration of neutrophils was slightly elevated (4.7 ± 3.4 /10^3^/LL) and it was significantly higher (*p* = 0.019) in the clinically suspected compared to confirmed group (mean difference 1.4 10^3^/LL). The mean concentration of C-reactive protein (CRP) was very high (30.2 ± SD 47.1 mg/l) with significantly higher mean concentration in clinically suspected (33.1 ± SD 47.8 mg/L) compared to confirmed (15.2 ± SD 40.4 mg/L) (*p* = 0.043) COVID-19 patients. The proportion of clinically suspected (69.7%) patients with an elevated CRP was 2.4 times higher compared with confirmed (28.6%) patients. On average, within 24–36 h of admission, the mean ferritin level elevated by 18.0 ± SD 100.3 μg/l, with an insignificant difference (*p* = 0.928) in the elevated ferritin level between COVID-19 clinically suspected and confirmed patients. Interestingly, the mean ratio of ferritin level rise was significantly (*p* = 0.049) higher in clinically suspected (mean ratio increased by 34%) compared with confirmed (mean ratio increased by 7%) patients. The ferritin level was doubled in 13 (11.8%) clinically suspected patients compared with only 1 (4.0%) confirmed patient after 24–36 h (Table 1). The mean bilirubin level was also significantly (*p* = 0.047) higher in clinically suspected (5.1 ± SD 3.7) compared with confirmed (4.0 ± SD 1.8) patients. The proportion of pediatric patients with at least one elevated inflammatory biomarker was significantly (*p* = 0.003) higher in clinically suspected (97.2%) compared with confirmed (83.3%) patients. Confirmed and clinically suspected COVID-19 patients were comparable in all other measured inflammatory biomarkers. Major abnormalities were seen in the chest X-ray of 82.2% of all pediatric patients with an insignificant difference in the chest X-ray examination between clinically suspected and confirmed COVID-19 patients (*p* = 0.159) (Table 2).

**Table 2:** Distribution of confirmed (RT-PCR-positive) and clinically suspected (RT-PCR-negative) pediatric patients according to their laboratory findings.

**Laboratory testing**	**All** ***n* = 173 (valid %)**	**Confirmed COVID-19** ***n* = 28 (valid %)**	**Clinically suspected** ***n* = 145 (valid %)**	***P*-value**
**Blood count**				
Mean leucocytes ± SD (10^3^/LL)	11.8 ± 29.7	8.7 ± 2.8	12.4 ± 32.4	0.549
Mean neutrophils ± SD (10^3^/LL)	4.7 ± 3.4	3.5 ± 2.7	4.9 ± 3.4	0.019
Mean lymphocytes ± SD (10^3^/LL)	11.7 ± 29.7	4.3 ± 2.2	3.7 ± 2.4	0.277
Mean platelet ± SD (10^9^/L)	286.6 ± 98.4	306.3 ± 87.9	282.8 ± 100.0	0.248
Mean hemoglobin ± SD (g/l)	11.9 ± 1.1	12.0 ± 1.3	11.9 ± 1.1	0.722
**Inflammatory biomarkers, mg/L**				
CRP, mean ± SD	30.2 ± 47.1	15.2 ± 40.4	33.1 ± 47.8	0.043
Normal (≤ 4)	64 (37.0)	20 (71.4)	44 (30.3)	<0.001
Elevated (>4)	109 (63.0)	8 (28.6)	101 (69.7)	
PCT–mean ± SD	0.8 ± 4.7	0.2 ± 0.39	0.9 ± 5.2	0.515
*Missing*	*65*	*7*	*58*	
LDH, mean ± SD	321.2 ± 89.6	313.9 ± 87.1	322.7 ± 90.4	0.649
Normal (≤ 250 U/L)	30 (20.7)	5 (19.2)	25 (21.0)	
Elevated (>250 U/L)	115 (79.3)	21 (80.8)	94 (79.0)	
*Missing*		*2*	*26*	
Ferritin at presentation, mean ± SD	112 ± 185.2	164.7 ± 413.0	101.3 ± 75.5	0.442
Normal ≤ 300 μg/L	143 (96.6)	24 (92.3)	119 (97.5)	0.212^a^
Elevated > 300 μg/L	5 (3.4)	2 (7.7)	3 (2.5)	
*Missing*	*25*	*2*	*23*	
Ferritin after 24–36 h, mean ± SD	134.0 ± 248.8	182.9 ± 546.7	123.1 ± 100.5	0.591
Normal ≤ 300 μg/L	127 (93.4)	24 (96.0)	103 (92.8)	0.481^a^
Elevated > 300 μg/L	9 (6.6)	1 (4.0)	8 (7.2)	
*Missing*	*37*	*3*	*34*	
Difference in mean ferritin ± SD	18.0 ± 100.3	16.4 ± 146.5	18.4 ± 87.4	0.928
Mean ratio of ferritin level	1.29 ± 0.93	1.07 ± 0.45	1.34 ± 1.00	0.049
Doubled ferritin level after 24-36 h				0.222^a^
Yes	14 (10.4)	1 (4.0)	13 (11.8)	
No	121 (89.6)	24 (96.0)	97 (88.2)	
*Missing*	38	3	35	
D-dimer, mean ± SD	0.82 ± 1.26	0.78 ± 1.20	0.83 ± 1.27	0.552^a^
Normal (≤ 1 mg/l)	113 (81.9)	20 (83.3)	93 (81.6)	0.839
Elevated (> 1 mg/l)	25 (18.1)	4 (16.7)	21 (18.4)	
*Missing*	35	4	31	
**Liver function**				
SGPT, mean ± SD u/l	21.0 ± 32.1	18.6 ± 13.5	21.5 ± 34.9	0.689
Bilirubin, mean ± SD mol/l	4.87 ± 3.4	4.0 ± 1.8	5.1 ± 3.7	0.047
Albumin, mean ± SD g/l	45.3 ± 3.3	46.7 ± 3.7	45.0 ± 3.2	0.056
Inflammatory biomarkers (LDH, CRP, first ferritin, D-dimer)				0.020
None elevated	7 (5.3)	4 (16.7)	3 (2.8)	
Only one elevated	51 (38.9)	12 (50.0)	39 (36.4)	
Only two elevated	53 (40.5)	5 (20.8)	48 (44.9)	
Only three elevated	17 (13.0)	2 (8.3)	15 (14.0)	
All elevated	3 (1.7)	1 (4.2)	2 (1.9)	
At least one elevated	124 (94.7)	20 (83.3)	104 (97.2)	0.006
*Missing*	*42*			
Inflammatory biomarkers (LDH, CRP, second ferritin, D-dimer)				0.003
None elevated	6 (5.0)	4 (17.4)	2 (2.1)	
Only one elevated	46 (38.3)	12 (52.2)	34 (35.1)	
Only two elevated	46 (38.3)	6 (26.1)	40 (41.2)	
Only three elevated	19 (15.8)	0 (0.0)	19 (19.6)	
All elevated	3 (2.5)	1 (4.3)	2 (2.1)	
At least one elevated	114 (95.0)	19 (82.6)	95 (97.9)	0.002
*Missing*	53	5	48	
Chest X-ray				0.313
Normal	19 (11.2)	2 (7.1)	17 (12.1)	0.159
Mild abnormalities seen	11 (6.5)	4 (14.3)	7 (5.0)	
Major abnormalities seen	139 (82.2)	22 (78.6)	117 (83.0)	

a *P-values estimated from the Fisher's exact test; SD, standard deviation*.

### Medication, Duration of Hospitalization, and Clinical Outcomes

Oxygen was needed by the majority of confirmed (96.4%) and clinically suspected (95.9%) COVID-19 patients but the mean duration of oxygenation was statistically longer in confirmed (5.8 ± 2.4 days) compared to clinically suspected (3.5 ± 1.4) patients (*p* < 0.001). The majority of patients were given a combination of antibiotics (95.9%) and bronchodilators (96.5%) with an insignificant different between both confirmed (92.6 and 96.4%, respectively) and clinically suspected (96.6 and 96.6%, respectively) patients. On average, the pediatric patients were hospitalized for 4.1 ± 2.5 days with 1.83 times longer duration of hospitalization for confirmed (6.6 ± 4.6 days) compared with clinically suspected (3.6 ± 1.4 days) patients (*p* = 0.002). Overall, 78.6% and 43.4% of confirmed and clinically suspected patients were hospitalized for ≥ 4 days (*p* = 0.001), respectively. Most of the patients (91.3%) stayed in a regular ward; only one confirmed patient was admitted to the pediatric intensive care unit (PICU) with no deaths recorded (Table 3).

**Table 3:** Distribution of confirmed (RT-PCR-positive) and clinically suspected COVID-19 (RT-PCR-negative) pediatric patients according to medications used, duration of hospitalization, and clinical outcome.

	**All** ***n* = 173 (valid %)**	**Confirmed COVID-19** ***n* = 28 (valid %)**	**Clinically suspected** ***n* = 145 (valid %)**	***P*-value**
**Oxygen requirement**				0.889
Yes	166 (96.0)	27 (96.4)	139 (95.9)	
No	7 (4.0)	1 (3.6)	6 (4.1)	
Mean days of oxygenation ± SD	3.9 ± 1.8	5.8 ± 2.4	3.5 ± 1.4	<0.001
**Antibiotics use**				0.339
Single	6 (4.0)	2 (7.4)	5 (3.4)	
Combination	165 (95.9)	25 (92.6)	140 (96.6)	
**Bronchodilators use**				0.956^a^
Yes	167 (96.5)	27 (96.4)	140 (96.6)	
No	6 (3.5)	1 (3.6)	5 (3.4	
Duration of hospitalization – mean	4.1 ± 2.5	6.6 ± 4.6	3.6 ± 1.4	0.002
± SD				
1–3 days	88 (50.9)	6 (21.4)	82 (56.6)	0.001
≥4 days	85 (49.1)	22 (78.6)	63 (43.4)	
**Antiviral used**				
Hydroxychloroquine	4 (2.3)	4 (14.3)	0 (0.0)	0.001^a^
Favipavir	1 (0.6)	1 (3.6)	0 (0.0)	0.162^a^
Lopinavir/ritonavir (Kaletra)	1 (0.6)	0 (0.0)	1 (0.7)	0.838^a^
Needed chest physiotherapy	157 (90.8)	27 (96.4)	130 (89.7)	0.228^a^
**Outcome**				
Regular care	158 (91.3)	27 (96.4)	131 (90.3)	0.263
High dependency	0	0	0	–
PICU	1 (0.6)	1 (3.6)	0	0.162
Death	0	0	0	
*Missing*	*15*		*14*	

a *P-values estimated from the Fisher's exact test; SD, standard deviation; PICU, pediatric intensive care unit*.

### Factors Associated With Severity of Disease at Presentation

In crude analysis, age in months (OR: 0.97, 95% CI: 0.96–0.99) and hemoglobin level (OR: 0.64, 95% CI: 0.45–0.90) were inversely associated with disease severity (*p* < 0.05), however, this inverse association disappeared in the confounders-adjusted model. Lymphocyte count (OR: 1.31, 95% CI: 1.12–1.53), platelets count (OR: 1.01, 95% CI: 1.00–1.01), LDH (OR: 1.01, 95% CI: 1.00–1.01), D-dimer (OR: 1.92, 95% CI: 1.28–2.88), and SGPT (OR: 1.04, 95% CI: 1.00–1.09) were all positively associated with presenting with a severe stage of the disease. Statistically insignificantly, pediatric patients with an elevated ferritin level (>300 μg/L) had 3.8 times higher odds of presenting with a severe stage of the disease (OR: 3.84, 95% CI: 0.61–24.39). Severity of the disease at presentation was significantly associated with an elevated ferritin level after 24–36 h (OR: 9.25, 95% CI: 2.23–38.31). Severity and elevated ferritin level after 24–36 h were the only positively correlated factors in the confounders-adjusted model (aOR: 17.38, 95% CI: 1.19–253.34). Clinically suspected or confirmed COVID-19 status was not associated with disease severity (Table 4).

**Table 4:** Bivariate and multivariate analysis of factors associated with severity of disease at presentation (severe vs. non-severe) among confirmed and clinically suspected COVID-19 pediatric patients.

**Characteristics**	**Crude OR (95% CI)**	**Adjusted OR (95% CI)**
Age in months	0.97 (0.96–0.99)^**^	0.99 (0.95–1.02)
Age, years	0.73 (0.60–0.90)^**^	–
>3 vs. ≤ 3 years	0.37 (0.15–0.90)^*^	–
Gender (female vs. male)	1.55 (0.72–3.33)	–
Residence status (citizen vs. resident)	1.35 (0.63–2.93)	–
Duration of symptoms (>3 vs. ≤ 3 days)	1.59 (0.73–3.46)	–
Contact with COVID-19 case (yes vs. no)	0.57 (0.19–1.77)	–
Confirmed vs. clinically suspected COVID-19	0.66 (0.21–2.05)	0.000 (0.000-NA)
Blood count	
Leucocytes count	0.99 (0.98–1.02)	–
Neutrophils count	0.91 (0.81–1.04)	–
Lymphocytes count	1.31 (1.12–1.53)^***^	1.29 (0.91–1.84)
Platelet count	1.01 (1.00–1.01)^**^	1.00 (0.99–1.01)
Hemoglobin	0.64 (0.45–0.90)^**^	0.50 (0.21–1.18)
Cytokines biomarkers		–
CRP	0.99 (0.98–1.01)	–
*Elevated: >4 vs. normal:*	0.76 (0.35–1.65)	–
* ≤ 4*		
PCT	0.78 (0.26–2.41)	–
LDH	1.01 (1.00–1.01)^**^	1.00 (0.98–1.01)
Ferritin level at presentation	1.00 (0.99–1.00)	–
*Elevated: >300 μg/L vs. normal: ≤ 300 μg/L*	3.84 (0.61–24.39)	–
Ferritin level after 24–36 h	1.00 (0.99–1.00)	
*Elevated: >300 μg/L vs. normal: ≤ 300 μg/L*	9.25 (2.23–38.31)^**^	17.38 (1.19–253.34)^*^
D-dimer	1.92 (1.28–2.88)^**^	
*Elevated*: >*1 mg/L vs. normal*: * ≤ 1 mg/L*	6.55 (2.43–17.64)^***^	2.61 (0.43–15.77)
Liver function	
SGPT	1.04 (1.00–1.09)^*^	1.05 (0.95–1.17)
Bilirubin	0.97 (0.82–1.14)	–
Albumin	1.05 (0.90–1.24)	–
Doubled ferritin level after 24-36 h (yes vs. no)	2.8 (0.8–10.1)	–

*OR, odds ratio. aOR, adjusted odds ratio for variables with significant association at P-value < 0.05 in the bivariate analyses in addition to the RT-PCR*.

* *P-value <0.05*,

** *P-value <0.01*,

*** *P-value < 0.001*.

### Factors Associated With Duration of Hospitalization

In the crude analysis, contact with a confirmed COVID-19 case increased the duration of hospitalization for one more day on average by 88% and by 280% (aOR: 3.80, 95% CI: 2.64–4.96) after adjusting for the COVID-19 status (confirmed or clinically suspected) and inflammatory biomarkers (hospitalization for ≥3 days: aOR: 6.8, 95% CI: 1.20–38.71). In the crude and adjusted analysis, both confirmed compared with clinically suspected (aOR: 4.00, 95% CI: 2.92–5.10) patients and patients with a moderate compared with mild stage of the disease at presentation (aOR: 5.87, 95% CI: 1.08–32.06) were also positively associated with longer duration of hospitalization (Table 5).

**Table 5:** Bivariate and multivariate analysis of factors associated with duration of hospitalization among confirmed and clinically suspected COVID-19 pediatric patients.

**Characteristics**	**Hospitalization in days**			**Hospitalization** (**≥3 vs**. ** <3 days)**		
	**Crude coefficient (95% CI)**	**Adjusted coefficient (95% CI)**		**Crude OR (95% CI)**	**aOR (95% CI)**	
		**Model 1**	**Model 2**		**Model 1**	**Model 2**
Age in months	0.01 (−0.00–0.02)	–	–	1.00 (0.99–1.01)	–	–
Gender (female vs. male)	0.26 (−0.50–1.01)		–	0.98 (0.54–1.77)	–	–
Residence status (citizen vs. resident)	−0.78 (−1.56 to −0.01)^*^	0.08 (−0.87–1.03)	0.22 (−0.39–0.84)	0.55 (0.29–1.03)	–	0.72 (0.21–2.43)
Duration of symptoms (>3 vs. ≤ 3 days)	0.29 (−0.51–1.08)	0.45 (-87–1.76)	–	0.77 (0.41–1.45)	–	–
Contact with COVID-19 case (yes vs. no)	1.88 (0.94–2.82)^***^	3.80 (2.64–4.96)^***^	0.35 (−0.82- 1.52)	9.77 (3.25–29.43)^***^	13.50 (0.69–261.43)	6.81 (1.20–38.71)^*^
Confirmed vs. clinically suspected COVID-19	2.99 (2.07-3.90)^***^	4.00 (2.92–5.10)^***^	2.25 (1.14–3.56)^***^	4.77 (1.83–12.47)^**^	18.32 (2.23–150.6)^**^	2.31 (0.27–19.83)
Severity of symptoms (Ref: mild)	−015 (−0.77–0.47)					
Moderate	–	–	–	4.86 (1.51–15.66)^**^	4.45 (0.83–23.83)	5.87 (1.08–32.06)^*^
Severe	–	–	–	2.00 (0.54–7.47)	0.81 (0.09–7.55)	0.95 (0.10–8.84)
Moderate/sever	–	–	–	4.00 (1.26–12.72)^*^	4.37 (0.77–24.75)	4.53 (0.68–30.07)
**Blood count**						
Leucocytes count	−0.01 (−0.02–0.01)	−1.69 (−2.88 to −0.50)	–	0.91 (0.85–0.98)^*^	0.87 (0.70–1.08)	0.96 (0.77–1.21)
Neutrophils count	−0.03 (−0.14–0.09)	1.91 (0.60–3.22)	–	0.89 (0.81–0.98)^*^	1.07 (0.79–1.45)	0.95 (0.68–1.33)
Lymphocytes count	−0.12 (−0.28–0.05)		–	0.94 (0.83–1.07)	–	–
Platelet count	0.003 (−0.001–0.01)		–	0.999 (0.99–1.01)	–	–
Hemoglobin ± SD g/dl	−0.13 (−0.46–0.20)		–	1.08 (0.83–1.40)	–	–
**Cytokines biomarkers**						
CRP	0.01 (0.00–0.02)	0.03 (0.01–0.034)^***^	0.004 (−0.004–0.01)	0.57 (0.31–1.07)	1.01 (0.99–1.02)	0.78 (0.17–3.60)
PCT	0.06 (−0.06–0.18)			2.75 (0.93–8.11)	–	3.41(0.66–17.79)
LDH	0.01 (0.00–0.01)^*^		0.00 (−0.004–0.003)	0.93 (0.41–2.08)	–	–
Ferritin at presentation	0.01 (0.008–0.01)^***^		0.004 (−0.001–0.01)	1.32 (0.21–8.15)	–	–
Ferritin after 24–36 h	0.008 (0.007–0.009)^***^		0.005 (0.001–0.008)^**^	2.76 (0.55–13.81)	–	–
Doubled ferritin level after 24–36 h (yes vs. no)	0.55 (−0.91–2.02)	0.80 (−0.53–2.135)	–	5.17 (1.11–24.09)^*^	6.10 (1.06–35.07)^*^	5.29 (1.03–27.21)^*^
D-dimer	0.09 (−0.27–0.45)	0.23 (−0.17–0.63)	–	0.71 (0.30–1.68)	1.73 (0.47–6.32)	–
**Liver function**						
SGPT	0.002 (−0.01–0.02)		–	1.02 (0.98–1.06)	–	–
Bilirubin	−0.13 (−0.27–0.01)	−0.15 (−0.27 to −0.03)^*^	−0.07 (−0.16–0.02)	0.83 (0.73–0.95)^**^	0.83 (0.70–0.97)^*^	0.90 (0.71–1.16)
Albumin	−0.16 (−0.30 to −0.02)^*^		0.08 (−0.02–0.18)	0.995 (0.90–1.11)	–	–
**Cytokines (LDH, CRP, first ferritin, D-dimer)**						
At least one cytokine elevated (yes vs. no)	−0.61 (−2.65–1.44)		–	0.47 (0.09–2.52)	–	–
Cytokines (LDH, CRP, second ferritin, D-dimer)			–			
At least one cytokine elevated (yes vs. no)	−0.772 (-3.03–1.48)		–	0.28 (0.03–2.43)	–	–
Antibiotic use (combination vs. single)	1.27 (−0.78–3.32)		–			
Bronchodilators use (yes vs. no)	1.28 (−0.77–3.33)	–		5.06 (0.58-44.26)	–	0.00 (0.00–NA)

*Model 1: adjusted for PCR-test, leucocytes, neutrophils, D-dimer, CRP, double ferritin level, bilirubin, and duration of symptoms*.

*Model 2: adjusted for residence status, contact with COVID-19 case, RT-PCR test result, CRP, LDH, ferritin at presentation, ferritin after 24–36 h, bilirubin, and albumin*.

* *P-value <0.05*,

** *P-value <0.01*,

*** *P-value < 0.001*.

## Discussions

The study discusses the differences in clinical presentation and laboratory findings of PCR-confirmed and clinically suspected COVID-19 pediatric patients. Also, the study identified factors associated with the severity of the disease and length of hospitalization among confirmed and clinically suspected COVID-19 pediatric patients. COVID-19-confirmed and clinically suspected pediatric patients were almost similar in all clinical and laboratory findings. Elevated ferritin level 24–36-h post-admission was independently associated with the severity of the disease at presentation. Confirmed COVID-19 patients were more likely to have a longer duration of hospitalization compared to clinically suspected patients. The study highlights the necessity of clinical evaluation and prompt management of children presenting with COVID-19 diagnostic criteria even if their RT-PCR is negative. This raises awareness that these children are at hidden risk of disease transmission to adults and the elderly, which could lead to a continuous viral spread.

In the initial phase of the COVID-19 outbreak, diagnosis of the disease was complicated by the diversity of symptoms and imaging findings, as well as the severity of the disease at the time of presentation (12). RT-PCR remains the gold standard of tests in confirming the diagnosis of COVID-19, however, it may still display an initial false-negative result (11). In the present study, only 16.1% of the children had a positive RT-PCR result for COVID-19. Earlier reports noted a false-negative rate of RT-PCR up to 30% (13). Failure to confirm a clear explanation for the presenting symptoms in our studied children with a negative RT-PCR result for COVID-19 supports the possibility that these children were COVID-19 patients, especially as 5.5% of these children had a documented history of contact with COVID-19 patients and clinically they were presenting COVID-19-like symptoms. We adopted the explanation that an RT-PCR false-negative may be related to the patient's viral load and virus shedding (13, 14) and the predominant site of viral replication (nasopharyngeal vs. lower respiratory tract) (15), in addition to the technical proficiency of the sample collectors and handlers. In this study, confirmed and clinically suspected COVID-19 pediatric patients demonstrated a similar sociodemographic status except for history of contact (more in confirmed cases) and residence status (more citizens). In a total of 82.1% of the confirmed cases, patients had a history of contact with an infected person with COVID-19. Previous studies showed that most of the infected children had a familial history of positive contact (16–19).

According to data from the International Severe Acute Respiratory and emerging Infection Consortium (ISARIC), a prospective multinational observational study, hospitalized children under 18 years of age who tested positive for SARS-CoV-2 experienced typical symptoms of fever (69%), cough (48%), and shortness of breath (23%), and 85% of children admitted had at least one of these symptoms (20). These are in concordance with our findings where fever and cough were the most commonly presented symptoms in our pediatric patients. Interestingly, our two groups (confirmed and clinically suspected cases) showed an insignificant difference in their reported symptoms. As described in previous reports, generally the degree of fever in our cases was <39°C (9, 21, 22).

The proportion of confirmed and clinically suspected COVID-19 pediatric patients with difficulty in breathing indicative of a lower respiratory tract infection was comparable. Moreover, pneumonia with significant chest X-ray findings was reported in 78.6% of confirmed cases and 83% of clinically suspected cases. These findings were more pronounced than a previous report in UAE where lower respiratory tract infection only counted for 9.7% (16) and was more common than in other reports (23, 24). The reasons for the significance of lower respiratory tract infection in our children could be attributed to the accepted admission criteria for COVID-19 patients to our facility.

In our study, only 7.1% of confirmed cases and 11.1% of clinically suspected cases had mild disease. Moderate illness was found in 78.6% and 66.0% while severe illness occurred in 14.3% and 20.1% of confirmed and clinically-suspected cases, respectively. These findings are consistent with earlier reported findings from different settings (6, 25, 26). However, the reason of the observed high proportion of moderate to severe cases in our study is unclear, this could be related to the disease activity and viral load, but we cannot rule out the potential bias in that our facility is a tertiary hospital and most of our children were admitted in a moderate to severe condition. Fortunately, none of our patients required critical care management which confirmed similar studies reporting a low rate of intensive care admission (12, 16, 22), where only one child was admitted to the PICU with a case of multisystem inflammatory syndrome in children (MISC) with Kawasaki-like features but with a positive RT-PCR test. Most of our children with lower respiratory tract infection required oxygen support but none of them suffered from acute respiratory distress syndrome reported in other studies (12, 27), or needed ventilatory support. This could be attributed to viral load and genetic, immunological, and environmental factors, moreover, the prompt management of these children from our side, whether confirmed or clinically suspected of COVID-19, improved their disease activity.

The laboratory findings of our study showed normal leukocyte, lymphocyte, and platelet levels. This was in contrast with other studies where lymphopenia was reported in 22.7% (22) and 16% (28). The overall neutrophil count was slightly elevated and more pronounced in the clinically suspected group compared to the confirmed group. These findings were consistent with other reports (21, 22, 27, 28) but in contrast to Elghoudi et al. who noted a high prevalence of neutropenia and thrombocytopenia in their studied population (16). The mean concentration of CRP (higher in the clinically suspected group) supported previous reports (22, 29, 30).

The reported duration of hospitalization in children with COVID-19 infection was variable. In our study, the average length of stay of confirmed COVID-19 pediatric patients was 6.6 ± 4.6 days, compared to other studies where the average length of stay was 3.3 days (16), 5.6 ± 4 SD days (23), and 11 days (31). The length of hospital stay in our confirmed group was longer in clinically suspected COVID-19 pediatric patients. The prolonged duration of hospitalization in our group could be attributed to higher incidence of moderate to severe cases in our study.

The present study has several potential limitations. The small sample size majorly contributes to the under-powered statistical estimates of association with the measured outcomes. Pediatric patients from a single center limits generalizability to other children in the country. Therefore, more large-scale and multicenter studies covering a large sample of symptomatic and asymptomatic pediatric patients are warranted. Another potential limitation is that the first negative PCR result was confirmed with a second PCR test for only 47% of pediatric patients. The unperformed second PCR test on the rest of the pediatric patients with a first negative PCR result could lead to a misclassification bias between PCR-confirmed and clinically suspected COVID-19 pediatric patients. However, failure to confirm a clear explanation for presenting COVID-like symptoms in our studied children with negative RT-PCR results for COVID-19 supports the possibility that these children were COVID-19 patients. Moreover, a blood culture, urine culture, respiratory viral screening panel for other possible viral causes of the presenting signs and symptoms including adenovirus, enterovirus, H1N1, respiratory syncytial virus, and other viral screening were all performed and reported as negative. Potential infection with mycoplasma was also excluded, and stool culture in children with gastrointestinal manifestations was normal. Despite these potential limitations, the implemented standardized methodology (PCR and laboratory testing) across all of the confirmed and clinically suspected COVID-19 pediatric patients reduces the potential risk of measurement and reporting bias between the two groups.

## Conclusion

In summary, this study has indicated that the main clinical features of confirmed and clinically suspected COVID-19 pediatric patients are fever, cough, and difficulty in breathing (mild pneumonia). In pediatric patients with negative RT-PCR results, COVID-19 is still clinically suspected based on clinical symptoms and epidemiological data, a tentative diagnosis can be made based on a thorough examination of laboratory test results and X-ray findings and children should be treated promptly. Larger clinical cohort and laboratory studies are required for better understanding of the possible implications of COVID-19 infection in children.

## Data Availability Statement

Data is subject to availability from the corresponding author upon justifiable reasons and after necessary official permission from concerned stakeholders.

## Ethics Statement

This study was approved by the Abu Dhabi Health COVID-19 Research Ethics Committee (IRB DOH/CVDC/2020/2417/IRB DOH/CVDC/2021/528). Patients' informed consent was waived given the retrospective nature of the study. Patients' medical records were only accessible by the investigator and co-investigator. Data was extracted without any identifiers (name, address, or telephone number). Patients' data was saved through a secure online database. Written informed consent from the participants' legal guardian/next of kin was not required to participate in this study in accordance with the national legislation and the institutional requirements.

## Author Contributions

NE, MS, and RA-R conceptualized the study and managed the project. NE, MS, BL, MO, LC, DM, and LE contributed to data curation, methodology, and data collection. RA-R performed the formal analysis and interpreted findings, contributed to the implemented methodology, and wrote the first draft of the manuscript. All authors reviewed and approved the final manuscript.

## Conflict of Interest

The authors declare that the research was conducted in the absence of any commercial or financial relationships that could be construed as a potential conflict of interest.

## Publisher's Note

All claims expressed in this article are solely those of the authors and do not necessarily represent those of their affiliated organizations, or those of the publisher, the editors and the reviewers. Any product that may be evaluated in this article, or claim that may be made by its manufacturer, is not guaranteed or endorsed by the publisher.
